# The Oxidative Stress Markers’ Protective Influence of Sea Buckthorn and Grape Extracts in Atorvastatin-Treated Hyperlipidemic Rats

**DOI:** 10.3390/nu16121954

**Published:** 2024-06-19

**Authors:** Romeo T. Cristina, Erieg A. Mohamed, Camelia Tulcan, Eugenia Dumitrescu, Florin Muselin, Sergiu A. Orășan, Teodora Mateoc-Sirb, Daliborca Vlad

**Affiliations:** 1 Faculty of Veterinary Medicine, University of Life Sciences “King Mihai I” from Timisoara, 300645 Timisoara, Romania; eriega.mohamed@gmail.com (E.A.M.); cameliatulcan@usvt.ro (C.T.); eugeniadumitrescu@usvt.ro (E.D.); florinmuselin@usvt.ro (F.M.); s.orasan@yahoo.com (S.A.O.); 2 Division of Biotechnology, Department of Applied Sciences, University of Technology, Baghdad 10066, Iraq; 3 Pharmacology Department, University of Medicine and Pharmacy “Victor Babes”, 300041 Timisoara, Romania; vlad.daliborca@umft.ro

**Keywords:** hyperlipidemia, oxidative stress, statins, plant extracts, murine model

## Abstract

Free radicals and reactive oxygen species initiate when the oxidative stress arises. (1) Background: The effect of natural molecules on oxidative stress in hyperlipidemic rats, taking statins, was observed. (2) Methods: One hundred and twelve white Wistar rats, males and females, were divided into seven: Group I received 20 mg of atorvastatin while groups II and III received a combination of 20 mg of atorvastatin and 100 mg of Sea buckthorn and grape extract. Groups IV and V received 100 mg of Sea buckthorn and grape extract, while groups VI and VII received only high-fat diet (HFD) and normal rodents’ fodder. After two and six months, rats were euthanized, and blood was gathered to measure the main paraclinical values and total antioxidant capacity (TAC). Also, the liver and kidney were stored for the organs’ cytoarchitecture. For statistics, two-way analysis of variance (ANOVA), was performed. (3) Results: HFD produced hyperlipidemia, accompanied by augmented serum and hepatic oxidative stress markers, in addition to a reduction in antioxidant enzyme activities and glutathione levels. Polyphenolic substances proven efficient against HFD caused oxidative stress. (4) Conclusions: Atorvastatin heightened the histological injuries caused by the fatty diet, but these were diminished by taking atorvastatin in combination with 100 mg/kg of plant extracts.

## 1. Introduction

Oxidative stress is a state that occurs when the oxidant and antioxidant systems of a living body are disrupted, leading to the production of reactive oxygen species (ROS) as well as free radicals [1,2]. The fatty diet increases oxidative stress by increasing ROS production. Elevated liver markers, inflammatory cytokines, and hypercholesterolemia are all symptoms of oxidative instability that lead to various diseases and disorders [3,4]. 

The modern lifestyle, which includes unhealthy food, is closely linked to oxidative stress through elevating lipid peroxidation and protein carboxylation, while decreasing the antioxidant system [5,6].

Both in human and animal models, hyperlipidemia related to a fatty diet has been demonstrated to be the main cause of atherosclerosis. ROS and the oxidation of LDL can lead to oxidized LDL [7]. Studies have ascertained high levels of MDA (malondialdehyde), high levels of serum ROS, and lipid peroxidase in human and animal models fed with fatty diets [6,8,9]. 

The first line of defense system against harmful substances and ROS are the antioxidant enzymes, where the superoxide dismutase (SOD), catalase (CAT), and glutathione peroxidase (GPx) are also considered the most important defense enzymes [10,11].

Studies have been conducted to investigate the beneficial effects of lipid-lowering drugs, especially with the statins group, and a published meta-analysis of statin therapy shows that the absolute risk reductions of treatment with statins were only 1.3% and 0.4% for myocardial infarction and stroke, respectively [12]. 

Atorvastatin belongs to a group of medicines called statins. It has been reported that some items from the statins group can serve as free radical scavengers and control ROS [13,14,15]. Authors are reporting that the percentage of patients in whom elevation in transaminases occurs secondary to atorvastatin treatment is about 1–3%. However, statins, like other drugs, have certain side effects, including neurological and neurocognitive effects, reproductive and gastrointestinal issues, hepatotoxicity, renal and urogenital toxicity, muscle-related adverse events, diabetes mellitus II, and, possibly, hemorrhagic stroke as well as hyperuricemia and myositis [13,14,15,16,17].

Nevertheless, it is not clear which is the percentage of patients having their atorvastatin treatment (and statin treatment in general) ceased due to soaring transaminases, but there is an undisputable (and probably growing) reticence in the general population regarding statin treatment relating to liver and kidney side effects; hereafter, the usefulness of finding therapeutic agents that might alleviate statins’ associated liver/kidney injuries is considered [16,17,18,19,20,21]. 

This disturbance leads to a change in the biomolecules’ functions, the permeability of the cellular membrane, and the alteration of cellular processes. Many processes, such as redox signaling, are included by free radicals. In addition to phagocytes, granulocytes and macrophages particularly play a role in the intracellular killing of pathogens [18,19,20,21]. 

When present in low to moderate quantities, ROS are vital in managing activities such as homeostasis support, and a variety of cellular functions [21]. 

The destruction of highly reactive substances alters the oxidative balance of metabolic syndrome and related conditions, causing cell and tissue malfunction and developing additional metabolic diseases. Promoting cell function and tissue homeostasis, reducing inflammation, as well as preventing chronic metabolic disorders all require a healthy diet. As a result, dietary changes have been proposed as a method of treating or preventing some metabolic illnesses [22,23]. 

Various foods and dietary patterns may have several impacts on health due to the diversity of energy and nutrients. For this purpose, plant extracts seem to diminish atorvastatin’s liver detrimental effects. As a solution, the phytochemicals’ efficacy to reduce hyperlipidemia and oxidative stress has been intensely explored, with the medicinal plants’ antioxidant properties being discovered [24]. 

Plants contain various natural substances as polyphenolic compounds that can improve health, and minimize and protect in metabolic syndromes, by improving the organs’ function, such as the liver and kidney. Because of the phenolic compounds, Sea buckthorn and grape extracts are considered valuable bio alternatives, being considered as polyphenol-rich sources [24,25,26,27,28,29,30,31,32].

The Sea buckthorn (*Hippophae rhamnoides* L.) (SBT) belongs to the *Elaeagnaceae* family. SBT has been used in various medicinal and nutritional applications. Bioactive chemicals are rich in this plant’s berries, seeds, leaves, and bark. SBT berries are high in vitamins A, C, E, and K, as well as carotenoids, flavonoids, and organic acids [33,34]. 

On the other hand, organic grapes contain known phenolic components, primarily anthocyanin and resveratrol, and have valuable antioxidant and anti-inflammatory features, being hypolipidemic and decreasing cell injury agents [33,34,35,36,37]. 

Certain studies confirmed that SBT and grape extracts have certain hypolipidemic properties in combination with atorvastatin in rats nourished with a high-fat diet (HFD) for six months of treatment [35]. In this aim, the primary goal of this study was to assess the protective effect of STB and grapes extract on oxidative stress markers. 

## 2. Materials and Methods

### 2.1. Ethics Commission Approbation

The study was approved by the Bioethics Committee of USAMVB Timișoara, Romania, under No. 6/30.01.2018, being in accordance with all domains’ national and international relevant regulations, all methods being reported in accordance with ARRIVE guidelines. Rats were placed for seven days before beginning to adapt to laboratory settings in agreement with the UE Directive 2010/63/EU, upon the treatment of animals used for research purposes [38,39].

### 2.2. Animals and Experimental Design

Rats were gathered from the accredited bio-base of the “Cantacuzino” National Research and Development Institute for Microbiology and Immunology Bucharest, Romania (NIRDMI). To assess the beneficial effects of phytotherapy with respect to hyperlipidemia initiated by a high-fat diet, white Wistar rats were randomly selected; 112 males and females, weighting from 150 g to 165 g, and aged 3 to 4 months, were included in research and divided into seven equal groups (n = 16), as shown in (Table 1). 

The rats were housed in regular cages (l × w × h = 750 × 720 × 360 mm) and fed ad libitum with the usual diet destined for rodents (Diet, Biovetimix, code 140-501, Romania). The temperature in the bio-base was 22 ± 2 °C and the relative humidity was 55 ± 10%. The animals were exposed to a 12/12 h light–dark cycle. 

**Scheme 1:**
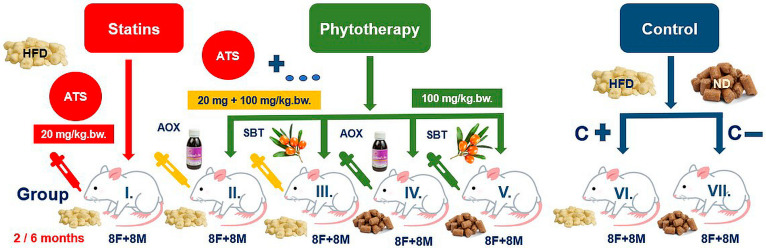
Experimental protocol organization. Where: HFD = high-fat diet, ATS = atorvastatin, SBT = Sea buckthorn *(Hippophae rhamnoides* L.) extract, AOX = Antioxivita—organic grapes extract, C+; C- = positive and negative control group.

After seven days of initial observation, to induce progressively hyperlipidemia, rats from Groups I, II, III, and VI were fed with an HFD recipe, initially presented by Doucet et al. (1987), and Group IV (organic grape extract), V (SBT extract), and Group VII (negative control) received only regular food for rodents (Diet, Biovetimix, Romania) and no HFD recipe [40]. Scheme 1 represents the experimental protocol.

Following two and, respectively, six months of therapy, all rats were euthanized, in agreement with known methodology, by injecting 300 mg/kg of body weight ketamine and 30 mg/kg.bw of xylazine, and blood samples were taken [38,39]. 

The serum was isolated and kept at −80 °C, then utilized to test antioxidant indicators. 

Metabolic organs (liver and kidney) were collected and held at −80 °C to assess the cytoarchitecture and evaluate the values of oxidative stress markers.

### 2.3. Statin Used in the Study

Atorvastatin (C_33_H_35_FN_2_O) (Sortis, Pfizer Europe, Sandwich, UK), molecular weight 558.65 g/mol, belongs to the group of statins. This structure belongs to the diphenylpyrrols, comprising heterocyclic aromatic molecules with a pyrrole ring attached to two phenyl groups. Atorvastatin is a 3-hydroxy-3-methylglutary CoA (HMG-CoA) reductase inhibitor, acting in hyperlipidemia, being the most used structure to reduce cholesterol levels (by lowering LDL via inhibiting HMG-CoA reductase) and prevent stroke by acting as an anti-inflammatory with other related mechanisms [14,41].

The statin administration in rats followed the same dosage recommended by the manufacturer per bodyweight in humans; therefore, the dose of 20 mg/kg body weight (which is considered the lowest therapeutic dose level in humans) was calculated [15,16].

In the experiment, statins were administered to the following groups: I (20 mg/kg.bw), II (20 mg/kg.bw + 100 mg/kg.bw organic grape extract), and III (20 mg/kg.bw + 100 mg/kg.bw SBT extract), as illustrated in Scheme 1. 

### 2.4. The Studied Plants 

#### 2.4.1. Organic Grape Extract (Antioxivita)

A commercial concentrated organic grape (*Vitis vinifera*) extract, labeled Antioxivita (Phenalex, Oradea, Romania) (SNPMAPS 9038), considered the most powerful natural antioxidant on the Romanian market, and obtained from certified organic farming, was gathered from an herbal pharmacy.

In conformity with the product information sheet, Antioxivita is a 100% natural, concentrated formula from grape skins, seeds, and rachis, and it was used in the study due to its proven phytochemical structure with tested high antioxidant potential. Antioxivita was obtained through a cutting-edge patented method, which succeeds in extracting and stabilizing antioxidants from grapes, in a high concentration to total polyphenol content (phenolic acids, anthocyanins, flavonoids, tannins, catechins, and resveratrol of 300 mg/mL GAE (equivalent to Gallic Acid)) (see Supplementary File S1).

The organic grape extract was administered at a dose of 100 mg/kg.bw to Group IV, or in combination with 20 mg/kg.bw of atorvastatin in Group II, as the experimental protocol shows. The HFD rats from Group VI served as positive control. Group VII served as negative control, receiving only a normal diet for rodents (Diet, Biovetimix, Romania).

#### 2.4.2. Sea Buckthorn Extract (*Hippophae rhamnoides* L.)

Sea buckthorn berries were chosen because of their large concentration of compounds with antioxidant, hypolipidemic, and therapeutic values [33,34]. The berries were purchased from a local herbal pharmacy. For preparing the SBT extract, an earlier modified approach was followed [35]. The amount was calculated to 100 mg/total polyphenol as one administration/day dosage. SBT extract was administered by gavage to rats at a dose of 100 mg/kg.bw, alone for Group V or in combination with 20 mg/kg.bw of atorvastatin in Group III. 

The total polyphenol content was determined using the modified Folin–Ciocalteu technique, with the evaluation of polyphenols content for Sea buckthorn and organic grape extract being presented in Supplementary File S1.

### 2.5. Biochemical Tests, Kits, and Reagents

#### 2.5.1. Samples Preparation for Serum

Colorimetric assay kits, Catalog No.: E-BC-K025; E-BC-K019; E-BC-K031; E-BC-K096; E-BC-K097, and, respectively, E-BC-K136-M (Elabscience Bionovation Inc., Houston, TX, USA), were used to measure the values of the serum and organs for MDA, T-SOD, CAT, GPx, T-GSH/GSSG, and TAC (total antioxidant capacity).

Fresh blood samples were taken for MDA, T-SOD, CAT, GPx, and TAC and clotted at 25 °C for 30 min. The extracted serum was preserved at −80 °C after being centrifuged at 2000 rpm for 15 min at 4 °C. The serum was prepared as usual for the measurement of T-GSH, GSSG, and GSH, then 400 μL of reagent working solution was added to 100 mL of a serum sample, well mixed with a vortex mixer for 30 s, then allowed to stand for 5 min [35]. After that, the mixture was centrifuged for 10 min at 3100 rpm at 4 °C. The supernatant was removed and analyzed. Following the manufacturer’s recommendations, the activity of MDA and antioxidant indicators was obtained.

#### 2.5.2. Tissue Homogenates Preparation

All procedures were carried out following the manufacturer’s instructions. First, 0.02–1.0 g of frozen tissue (−80) °C was washed thoroughly with PBS (Phosphate Buffer Solution) (0.01 M, pH 7.4) at 2–8 °C to remove blood cells for assessing the values of the MDA, T-SOD, and CAT. Then, water was absorbed with filter paper and 0.1 g of tissue was weighed and homogenized at the ratio of the volume of homogenized medium PBS (2–8 °C) mL to the weight of the tissue (g) of 9:1 (the 0.1 g was mixed with 0.9 mL of PBS in an ice bath using ultra sonication at 50% amplitude). The crude tissue homogenate was centrifuged at 10,000 rpm for 10 min at 4 °C. The final supernatant was removed and stored on ice for detection on the following day. 

The same sample preparation protocols were used for GPx, but with a different homogenization medium, which consisted of 10 mM of TRIS-HCl (pH 7.4), 10 mM of NaCl, 10 mM of sucrose, and 10 mM of EDTA. The protein concentration was measured using an RxDaytona Biochemistry analyzer (Randox Laboratories, Crumlin, UK). A small tissue specimen was captured and washed using normal saline, absorbing the water on the surface of the tissue. Afterward, 0.05 g was added to 0.45 mL of homogenized medium, then homogenized mechanically using laboratory mortar in an ice bath to prepare a 10% homogenate; the crude tissue homogenate was then centrifuged at 10,000 rpm for 10 min at 5 °C, and finally, the supernatant was picked for identification on the same day.

The spectrophotometric assessment of oxidative stress indicators and antioxidant variables is presented in depth in Supplementary File S1.

### 2.6. Histological Examination 

The tissue samples were set in 80% alcohol, for seven days, before being rinsed in distilled water and dehydrated by immersing in increasing concentrations of ethanol. Ethanol was substituted with xylene, and samples were then paraffin-hardened (Merck, Darmstadt, Germany). Slices of 5 mm were sectioned on a Cut-4062 microtome (Slee Medical GmbH, Nieder-Olm, Germany), mounted on slides, and stained with hematoxylin and eosin (H&E). For image interpretation, microscopy was performed at 20, 50, and 200 μm using an Olympus CX41 microscope (Olympus, Hamburg, Germany), which included a digital camera and QuickPhoto-Micro2.2 software (Promicra, Prague, Czech Republic).

### 2.7. Statistical Analysis

The SEM (Standard Error of the Mean) was employed. In order to determine whether data are normally distributed or not, initially a Shapiro–Wilk normality distribution test was used, followed by the difference and statistical significance analyses between groups, using the two-way ANOVA (analysis of variance) with Tukey’s multiple comparison test and Bonferroni correction, using Graph Pad Prism Software 9.0 (San Diego, CA, USA).

## 3. Results

### 3.1. Oxidative Stress Markers and Antioxidant Parameters

#### 3.1.1. Serum

The findings revealed a statistically significant elevation in MDA serum concentrations in Group VI in comparison to the control (*p* < 0.001). After two months and after six months of therapy, statin therapy showed a synergistic effect with 100 mg of phytotherapy resulting in a statistically significant reduction in MDA concentration compared to the control group (*p* < 0.001). 

**Figure 1:**
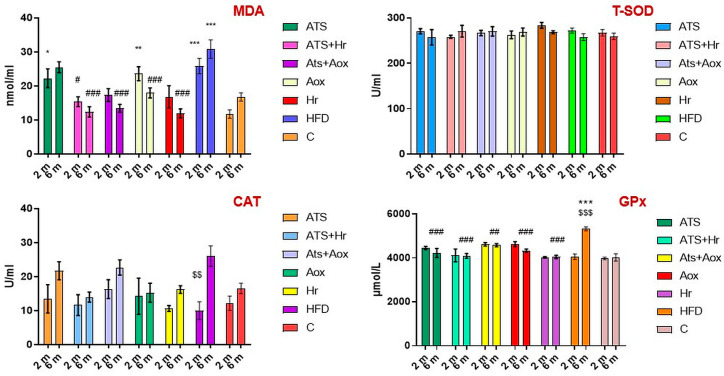
The activities of the serum antioxidant enzymes in the different experimental groups during the whole experimental period. Where: MDA (* meaning, *p* ˂ 0.05, ** meaning *p* ˂ 0.01, *** meaning, *p* ˂ 0.001 comparative to C; ^#^ meaning, *p* ˂ 0.05, ^###^ meaning *p* ˂ 0.01 comparative to HFD); CAT (^$$^ meaning, *p* ˂ 0.01 comparative two months to six months); GPx (*** meaning, *p* ˂ 0.001 comparative to C; ^##^ meaning, *p* ˂ 0.01, ^###^ meaning, *p* ˂ 0.001 comparative to HFD; ^$$$^ meaning, *p* ˂ 0.001 comparative two months to six months). Significance of the abbreviations shown in Figure 1, Figure 2, Figure 3, Figure 4, Figure 5 and Figure 6 is the following: ATS = atorvastatin; ATS + Hr *(Hippophae rhamnoides)* = atorvastatin + SBT extract; Ats + Aox = atorvastatin + organic grape extract (Antioxivita); Aox = organic grape extract; Hr = SBT; HFD = high-fat diet group; C = control group).

Following six months of treatment, the values of the antioxidant enzyme in Group VI exhibited a non-significant drop in serum T-SOD activity, whereas the CAT and GPx values were elevated compared to the values in the normal control group. Furthermore, phytotherapy considerably enhanced GPx values in treatment groups (*p* < 0.001) when compared to the normal control group (Figure 1).

An evaluation of the polyphenols content for Sea buckthorn and organic Grape extract and the values for oxidative stress markers and antioxidant parameters are presented in Tables S1–S6, in the Supplementary File S1.

**Figure 2:**
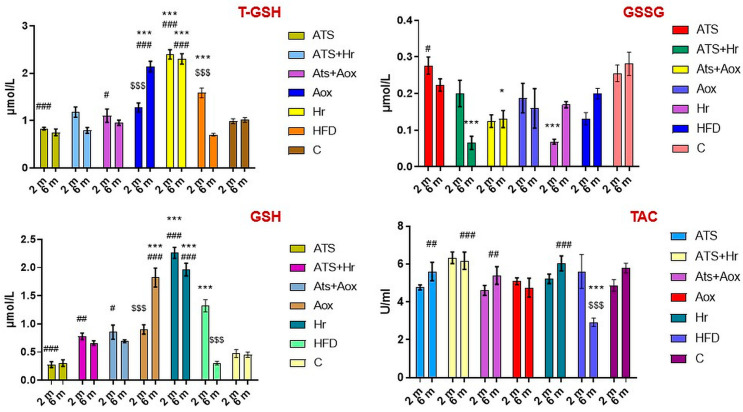
The activities of the serum antioxidant indicators in the different experimental groups during the whole experimental period. Where: T-GSH (*** meaning, *p* ˂ 0.001 comparative to C, ^#^ meaning, *p* ˂ 0.05, ^###^ meaning, *p* ˂ 0.01 comparative to HFD, ^$$$^ meaning, *p* ˂ 0.001 comparative two months to six months); GSSG (* meaning, *p* ˂ 0.05, *** meaning, *p* ˂ 0.001 comparative to C, ^#^ meaning, *p* ˂ 0.05 comparative to HFD), GSH (*** meaning, *p* ˂ 0.001 comparative to C, ^#^ meaning *p* ˂0.05, ^##^ meaning, *p* ˂ 0.01, ^###^ meaning, *p* ˂ 0.001 comparative to HFD, ^$$$^ meaning, *p* ˂ 0.001 comparative two months to six months); TAC (*** meaning, *p* ˂ 0.001 comparative to C, ^##^ meaning, *p* ˂ 0.01, ^###^ meaning, *p* ˂ 0.001 comparative to HFD, ^$$$^ meaning, *p* ˂ 0.001 comparative two months to six months).

The results revealed a statistically significant difference in the T-GSH and GSH levels in HFD in comparison to other treated groups. The T-GSH, GSH, and TAC levels improved significantly following six months of phytotherapy with SBT and grape extract in Groups IV and V, as represented in Figure 2.

#### 3.1.2. Liver

**Figure 3:**
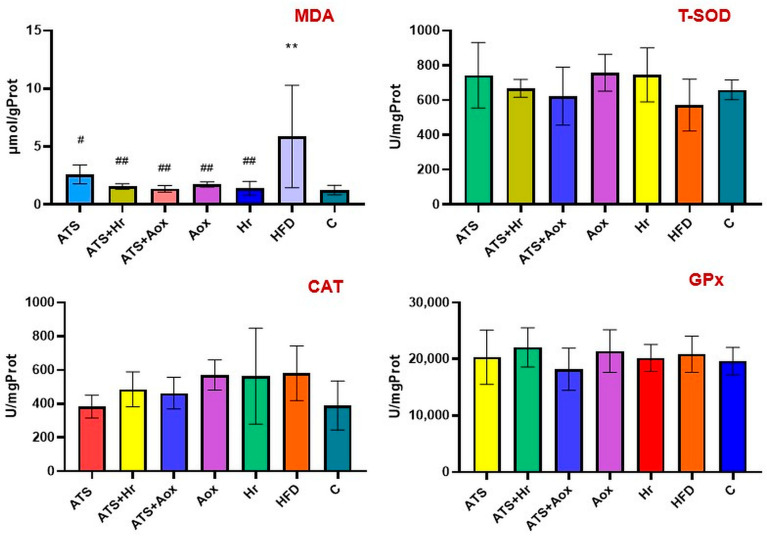
The hepatic MDA; T-SOD; CAT, and GPx activities in various experimental groups following six months of therapy. Where: ** meaning, *p* ˂ 0.01 compared to control; ^#^ meaning, *p* ˂ 0.05, ^##^ meaning, *p* ˂ 0.01 compared to HFD.

MDA is considered an important indicator for lipid peroxidation in hyperlipidemic conditions. The hyperlipidemic control group had much more lipid peroxidation than the normal group, as evidenced by higher MDA values. The use of atorvastatin alone or in conjunction with phytotherapy compounds considerably lowered the lipidic peroxidation indicator. The combination of atorvastatin and 100 mg of organic grape extracts inhibited lipidic peroxidation. When compared to the hyperlipidemic group, SBT dramatically reduced lipid peroxidation (*p* ˂ 0.01), as shown in Figure 3. 

The SOD concentrations in the control positive group were statistically lower than in the negative control group. Group I (atorvastatin) and Groups IV and V (only phytotherapy) showed elevated T-SOD levels statistically more than when they were supplemented with atorvastatin. In comparison to the fatty diet group, the treatment of phytotherapy resulted in the highest non-significant elevation in T-SOD concentration in Groups IV and V (*p* ˂ 0.01). 

Compared to the control group, the CAT values showed a non-significant elevation in the HFD rats. When the results were compared to control, atorvastatin did not affect CAT activity. Furthermore, atorvastatin in combination with phytotherapy elevates CAT readings statistically in comparison with control. 

**Figure 4:**
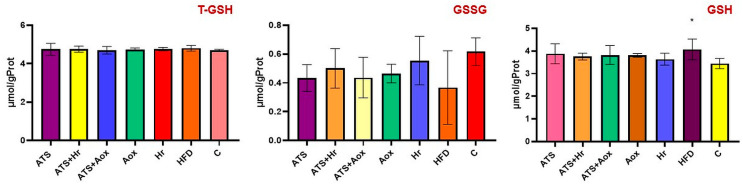
The hepatic T-GSH/GSSG activities in various experimental groups following six-month therapy. Where: * meaning, *p* ˂ 0.05 comparative to control.

Additionally, phytotherapy in Groups IV and V led to a slight improvement in CAT levels when compared to the control. Furthermore, there was no variation in GPx values across experimental groups. Concerning GSH, there was no statistically significant change in the T-GSH/GSSG levels between the HFD and regular diet groups (Figure 4). 

#### 3.1.3. Kidney

The results revealed no statistically significant changes in MDA or T-SOD levels. In comparison to the control negative group, a combination of 20 mg of atorvastatin and 100 mg of SBT showed the greatest improvement in CAT concentration (*p* ˂ 0.05). 

**Figure 5:**
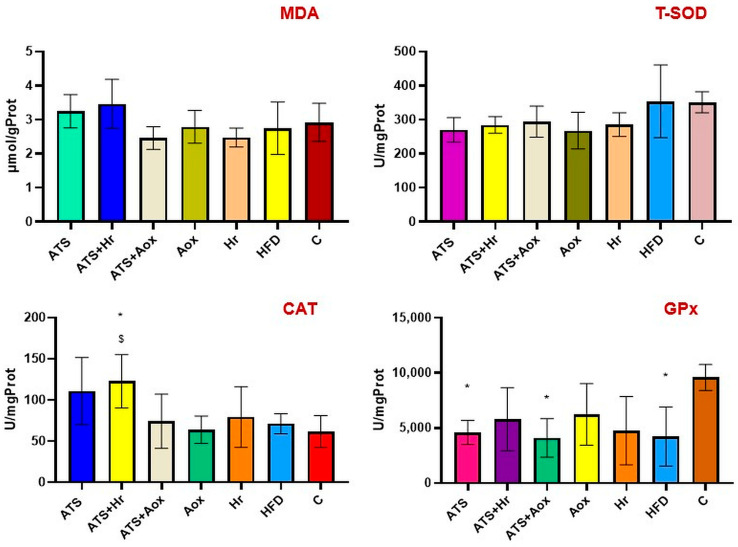
Renal MDA; T-SOD; CAT, and GPx activities in various experimental groups following six months of therapy. Where: CAT (* meaning, *p* ˂ 0.05 comparative to C, ^$^ meaning, *p* ˂ 0.05 comparative to grape extract); GPx (* meaning, *p* ˂ 0.05 comparative to control).

Following six months of treatment, there was a significant rise in the concentration of renal GPx in groups that received phytotherapy with SBT (Group II) and organic grape extract (Group IV) (*p* ˂ 0.05) (Figure 5). 

**Figure 6:**
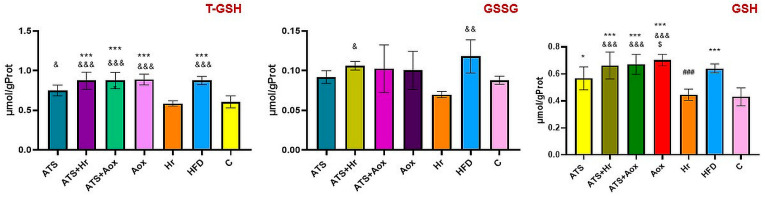
The renal T-GSH, GSSG, and GSH activities of different experimental groups after six months of therapy. Where: T-GSH (*** meaning, *p* ˂ 0.001 comparative to C; ^&^ meaning, *p* ˂ 0.05, ^&&&^ meaning, *p* ˂ 0.001 comparative to SBT); GSSG (^&^ meaning *p* ˂ 0.05, ^&&^ meaning *p* ˂ 0.01 comparative to SBT); GSH (* meaning, *p* ˂ 0.05, *** meaning, *p* ˂ 0.001 comparative to C, ^###^ meaning, *p* ˂ 0.001 comparative to HFD, ^$^ meaning, *p* ˂ 0.05 comparative atorvastatin; ^&&&^ meaning, *p* ˂ 0.001 comparative to SBT).

The results revealed a significant statistical elevation in GSH levels in the HFD group compared to the normal control group, while 100 mg of Antioxivita caused the largest rise in GSH values compared to the results from the normal control group (*p* ˂ 0.001) (Figure 6).

### 3.2. Histological Findings

As a general comment, it was found out that in great part, the most visible cytoarchitectural characteristics were noticed after six months of administration, both in the liver and kidney. The images after two months’ treatment were presented where cytoarhitectural modifications were perceived. 

#### 3.2.1. Liver Cytoarchitecture

The rat model of NAFLD has been created, according to H&E staining of liver isolates obtained from the HFD groups. The presence of lipid droplets in the cytoplasm of the hepatocytes, giving the cytoplasm a vesicular appearance, demonstrated the development of steatosis to varying degrees in the liver samples from Group VI, which received a rich fatty diet. After two months of administration, in comparison with a normal diet, the number of hepatocytes with a tiny vesicular aspect (ballooning degeneration) begins to develop. 

The fatty degeneration appears to be more severe six months after therapy than after two months. Male liver hepatocytes are more severely impacted than female liver hepatocytes. As a result, huge droplets of lipid collect inside the cytoplasm, pushing the nucleus to the cell’s periphery. 

Hepatocytes are large in size, resulting in hepatocellular ballooning with rarefied cytoplasm, most likely due to a change in the intermediate filaments from the cytoskeleton structure. A microscopic examination of the female rat after two months of atorvastatin therapy represented by Group I revealed the presence of irregular hepatocytes with vacuolar cytoplasm, pyknotic nuclei, and karyolysis among typical hepatocytes with homogeneous, compact cytoplasm and round, central nuclei. 

Additionally, some female liver samples exhibited very large hepatocytes, while others showed no lipid droplets. In males, steatosis was evident with large vesicular hepatocytes at the periphery of the hepatic lobules. However, other forms of liver injury were just beginning to appear compared to those on a normal diet. In some cases, normal hepatocytes with large nuclei were observed around the central veins.

The combination of atorvastatin with SBT and organic grape extract in Groups II and III demonstrated normal histological features of the liver architecture. In contrast to the HFD group, males have the most visible indicators of cellular recovery, particularly after six months of therapy. A histological section revealed no vesicular hepatocytes or inflammatory infiltration. A slight sinusoid capillary dilatation was also observed. 

**Figure 7:**
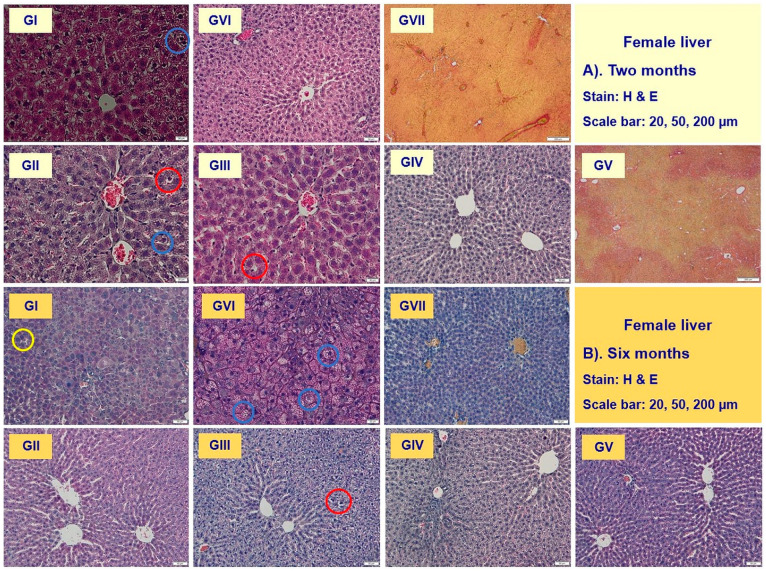
Histological section from female rat liver from experimental groups, after two and six months of treatment. Legend: ballooning degeneration low, mild, high ●; mild presence of pyknotic nuclei, vacuolar cytoplasm ●; large hepatocytes without droplets of lipids ●. Stain: H&E Scale bar: 20, 50, 200 μm.

**Figure 8:**
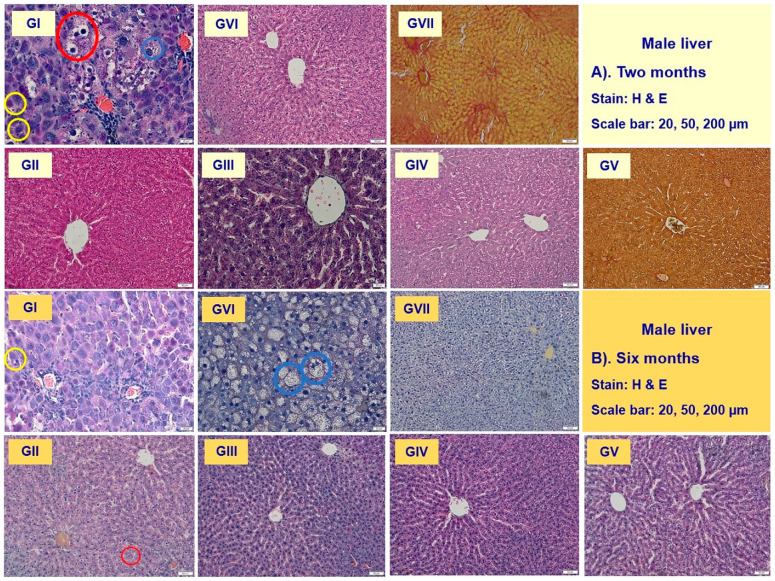
Histological section from male rat liver from experimental groups, after two and six months of treatment. Legend: ballooning degeneration low, mild, high ●; mild presence of pyknotic nuclei, vacuolar cytoplasm ●; large hepatocytes without droplets of lipids ●. Stain: H&E Scale bar: 20, 50, 200 μm.

A microscopic examination of the liver in Groups IV and V treated with organic grape extract and SBT, after two months, revealed the presence of tiny vesicular hepatocytes clustered in the periphery of the hepatic lobule in female rats, but an apparently normal section in males. Meanwhile, a histological investigation performed six months after treatment showed there was mild steatosis with ballooning hepatocytes (Figure 7 and Figure 8).

#### 3.2.2. Kidney Cytoarchitecture

The kidneys of the Group VI animals revealed vascular congestion as well as renal capsular space expansion. Some of the vascular glomeruli were destroyed after six months. Some nephrocytes have small vesicular aspects, while others became turgescent, which is hydropic degeneration. 

Adipocytes of a large size were found in the medullary zone of female rats. After two months of treatment with atorvastatin, the kidney appeared normal, but after six months, the renal capsular space was reduced in comparison to HFD, associated with vascular congestion. 

**Figure 9:**
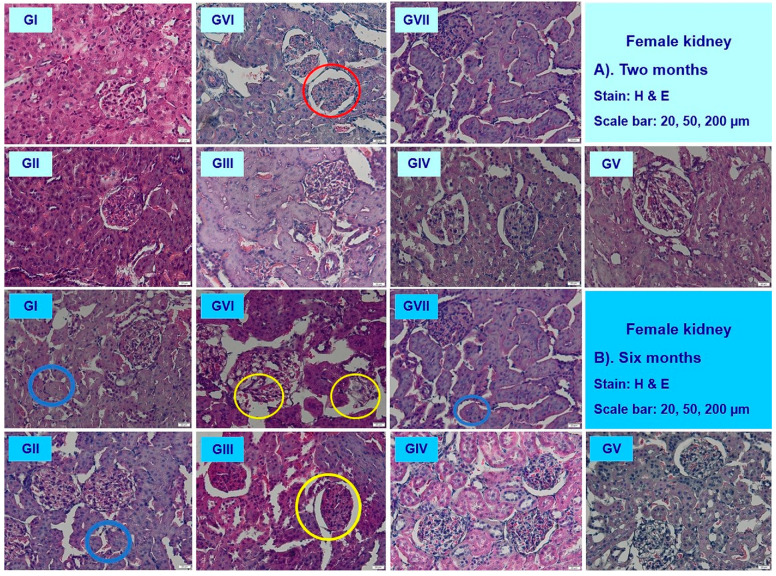
Histological section from female rat kidney from experimental groups, after two and six months of treatment. Legend: turgescent ●; enlarge space ●; destroyed vascular glomerulus ●. Stain: H&E Scale bar: 20, 50, 200 μm.

**Figure 10:**
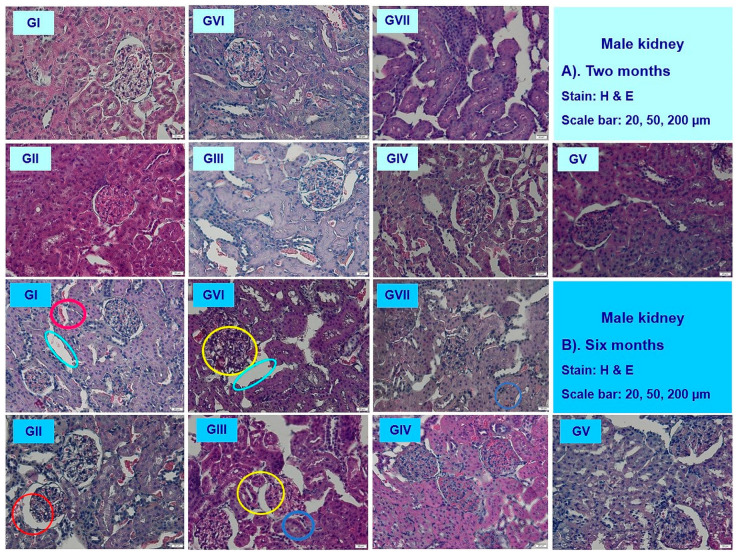
Histological section from male rat kidney from experimental groups, after two and six months of treatment. Legend: turgescent ●; enlarge space ●; destroyed vascular glomerulus ●; congestion ●; edema ●. Stain: H&E Scale bar: 20, 50, 200 μm.

The administration of atorvastatin significantly minimized the HFD changes, due to its potent antioxidant and lipid-lowering properties. After two months and after six months of treatment with atorvastatin, in combination with SBT or organic grapes extract, normal kidney function was exhibited, with minor signs of vascular congestion. After two months of treatment with organic grape extract (Group IV) and SBT (Group V), histological aspects were normal, with minor changes in vascular congestion and edema observed in some specific zones, particularly after six months of treatment, as shown in Figure 9 and Figure 10.

## 4. Discussion

### 4.1. Biochemical Results 

A fat-rich diet is considered the main cause that increases the chance of developing hyperlipidemia, which can lead to cardiovascular issues. In a 2003 study, feeding rats an HFD was proven to be a reasonable model for the possible consequences of dietary fat in human [42] and animal models [43]. 

Increased oxidative stress has been associated with HFD intake. Statistical data revealed significantly higher levels of MDA in the HFD group in serum blood and hepatic tissue, but no substantial changes were shown in renal tissues perhaps due to the short time frame of the study. This suggests that ROS may have induced cytotoxicity in the HFD group, but this is not clearly evident yet. 

Also, serum MDA values increased with time, suggesting an excess of free radical formation that resulted in lipid peroxidation and cellular damage, both of which have been connected to the development of hyperlipidemia complications. Also, it is important to consider not only the possibility of increased free radical production but also the potential decrease in antioxidant systems as contributing factors to the rise in serum MDA values [5,7,44].

The results obtained are in line with those of Yang et al., 2008 [7], who reported that MDA levels were higher in hyperlipidemic people with higher lipid levels. High levels of lipid peroxidation are thought to be caused by oxidative stress, which occurs when the dynamic balance between pro-oxidant and antioxidant processes fails [45]. Alterations in the physical properties of the cellular membrane have been associated with hyperlipidemia, which promotes free radical passage from the electron transport system or activates NADPH oxidase [46]. 

Also, it is possible that the cellular membrane could be affected by the type of fatty acids intake through diet. Monounsaturated fatty acids (MUFA) are less prone to oxidation than polyunsaturated fatty acids (PUFA), as authors observed [42,47]. 

The present study verifies the heterogeneity of lipid constants in murine models fed with HFD, as well as a control diet. The para clinic markers for an oxidative stress response could not be deduced reliably from MDA basal serum concentrations. For example, in the absence of therapeutics, research in hypercholesterolemic rabbits has also shown that lipid lowering through dietary management can reduce oxidative stress [48]. 

The circulating values of MDA in HFD serum and liver were 30.853 ±2.657 nmol/mL and 5.874 ± 1.806 μmol/g, respectively, in comparison to the normal diet group, indicating that the high values of MDA could be thought to be due to ROS overproduction and impairment in the antioxidant system. Moreover, lipid peroxidation concentrations fell dramatically in the phytotherapy groups, particularly in the SBT group, when compared to the HFD group, due to the abundant phenolic compounds in this plant [49].

The roles of the antioxidant defense system varied among the phytotherapeutic, fatty diet, and normal diet groups. T-SOD and GPx are crucial for clearing superoxide anions and hydrogen peroxide, providing cellular defense against oxidative damage as the first line of antioxidant defense. The insufficient detoxification of ROS by antioxidant enzymes can lead to an imbalance between antioxidant and oxidant systems. In vitro studies have shown that GPx is a relatively stable enzyme, with its levels changing in response to elevated oxidative stress [50,51,52,53].

The animal body has an efficient strategy for dealing with free radical-induced cell damage. Some of the powerful antioxidant enzymes involved in this process include T-SOD, CAT, as well as GPx, GSH, and GST [54,55,56]. 

The T-SOD activity levels in serum as well as in liver samples were found to be lower, but renal tissue showed a non-statistically significant alteration. While the serum and liver CAT readings of the HFD group exhibited no significant rise, the kidney CAT value did not alter following six months of treatment as compared to the control group. The non-significant rise in CAT enzyme concentration in the blood and liver tissues of HFD rats might be attributed to the liver tissue’s augmented oxygen consumption, caused by obesity, and the CAT enzyme concentration change which also is oxygen-dependent [50,51,52,53].

GPx levels are much lower in the HFD group’s renal tissue, but there is no influence in the liver tissues. GPx levels in serum are statistically higher in the hyperlipidemic group. The decrease in antioxidant enzyme values happens due to their utilization and storing exhaustion as a result of their role in battling free radicals collected during the initiation and progression [50,51,52,53].

CAT, T-SOD, and GPx concentrations were enhanced after grape and SBT intake. Glutathione plays a protective role by changing the redox condition of the cells and serving as an antioxidant enzyme mediator [50,51,52,53].

Findings from this study are consistent with Sehiril et al. (2008) who found that rats’ glutathione levels increased considerably after consuming grape seed extract. These findings were attributed to the polyphenols found in grapes, which are considered polyphenolic-rich plants [31].

Additionally, it is widely acknowledged that the polyphenols in the two plants studied have various essential anti-oxidative stress actions, such as the suppression of inflammation and prevention of LDL oxidation, and they have a protective role for the cells and tissues from oxidative stress injuries [33,34,35,36,37,54,55,56,57]. 

Therefore, polyphenols have strong antioxidant properties for scavenging reactive oxygen, which causes these effects. Phytotherapy alone or conjugated with 20 mg of atorvastatin improved the antioxidant enzymes’ state in experimental animals’ blood, hepatic, and renal tissue.

### 4.2. Histological Results 

The study demonstrated the development of fatty liver in rats, with typical histological features caused primarily by the impact of relatively large hepatocytes due to cumulative lipid compacting the sinusoidal lumen. Lipid droplets cause hepatic microcirculation damage as well as hepatocellular degeneration, fibrosis, and steatosis. Rat liver samples stained with hematoxylin and eosin showed steatosis injuries in hepatocytes. 

The findings are consistent with Buchner’s (2014) who observed that a high-fat diet causes lipid peroxidation, hepatocellular degeneration, and steatosis in rats’ livers [27].

In comparison with the control group, HFD reduced sinusoidal diameter, density, and blood flow. In addition, mixed micro vesicular and macro vesicular steatosis related to the size of accumulated fat vesicles within hepatocytes revealed a different picture of microcirculatory impairment related to the size of lipid droplets [16,58].

In NAFLD patients, per cellular and portal fibrosis, ballooning hepatocytes, and lobular inflammation have all been linked to oxidative stress and mitochondrial dysfunction. We observed that ballooning degeneration, microvilli enlargement, cell infiltration, and hepatocyte fibrosis were presented. These symptoms could be the result of oxidative damage in hepatocellular proteins and the necrotic changes in hepatocytes caused by HFD [59,60].

Hepatic steatosis tends to promote cellular tolerance for high levels of oxidative stress, permitting cells to survive in this toxic environment while remaining vulnerable to inflammatory processes such as apoptosis and necrosis. Fatty acid levels in hepatic tissue are high enough to damage cellular proteins and lipids, increase oxidative stress, and activate receptors associated with inflammatory hepatocellular lesions, defense cell activation, and tissue fibrosis [58,59,60]. 

Oxidative stress influences the pathophysiology of atorvastatin-induced light hepatotoxicity. The administration of atorvastatin to rats on a daily basis caused mechanisms of hepatic oxidative stress, cellular damage, and malfunction. Due to hepatic lipid peroxidation, statins can cause cellular damage in the liver, indicating that oxidative stress plays a role in atorvastatin-related hepatotoxicity. The findings are consistent with those of Farag [2015], who discovered that atorvastatin can cause hepatic lipid peroxidation and damage, implying that atorvastatin-induced hepatotoxicity is caused by oxidative stress [16].

The association of atorvastatin and SBT and grape extracts was beneficial, resulting in normal histological features of the liver architecture. In contrast to the HFD group, males have the most visible indicators of cellular recovery, particularly after six months of therapy. The phenolic and flavonoid composition of SBT and grape extract is mainly accountable for their antioxidant properties. They have been shown to reduce stress by limiting free radical oxidation by initiating or propagating the oxidizing chain reaction. 

The domain literature also supported these findings. Grape extract exacerbated the histological injury resulting from a high-fat diet, particularly when combined with pharmaceuticals such as atorvastatin. The abnormalities were lowered by consuming grape extract on a daily basis, which protected the liver from HFD-induced hepatocyte damage, revealing apparently normal hepatocytes compatible with a balanced diet [55,56,57].

A light microscopic examination showed blood vessel dilation and Bowman’s space dilation, in addition to cell infiltration, tubular defect, and nephrotic degeneration. An HFD causes rat obesity and may cause renal defects due to histological changes in the kidney, such as dilatation, tubular abnormalities, inflammation, and connective tissue enlargement. The results indicated that the HFD significantly increased inflammatory cells in the kidney histological analysis, which may be related to lipid deposition in the kidney. Furthermore, kidney volume enlargement could be attributed to edema caused by cell infiltration among the tubes. The results presented are in agreement with those of Altunkaynak et al. (2008) and Salim et al. (2018) [61,62].

Renal damage can be aggravated by oxidative stress caused by HFD due to LDL oxidation, which causes endothelial cell damage and the loss of normal kidney function [62]. 

After two months and after six months, treatment with atorvastatin in combination with SBT or organic grape extract demonstrated normal kidney function only with minor signs of vascular congestion. The phytotherapeutic properties of SBT and organic grape extracts can be attributed to the polyphenolic and active compounds found in these plants [25,28,56].

After two months of treatment with grape extract (Group IV) and SBT (Group V), histological aspects were normal, only with minor changes in vascular congestion and edema observed in specific zones, particularly after six months of treatment, and the results agree with those of Charradi et al. (2013), who found that grape seed and skin extract can protect rats given HFD for six weeks from HFD-induced renal lipotoxicity and other kidney-related illnesses [28].

## 5. Conclusions

Hyperlipidemia caused by a fatty diet is accompanied by elevated serum and hepatic oxidative indicators, as well as a decrease in the antioxidant parameters activities and glutathione values. The present study opens new opportunities in lessening statin-induced liver toxicity. At this stage, clinical studies on human subjects are essential to decide whether this approach might decrease the proportion of individuals interrupting treatment due to liver-associated side effects. 

The phytotherapeutics used are revealed to possess a beneficial hypolipidemic and hepatoprotective activity. The results established that polyphenolic substances, with or without atorvastatin therapy, can protect against oxidative stress caused by fat diets, the results being attributed mainly to their capacity to diminish the negative effect of ROS.

The study identifies the highest synergistic potential action for future use in the development of new drug designs or compositions (SBT extract and statin group, in the case of hyperlipidemia). The limitations of the study are related to the period which could be prolonged, with probably more accurate cytoarchitecture findings and the number or replicas of this study, which would be beneficial for the accurateness. Also, regardless of several studies on Sea buckthorn in various parts of the world, knowledge about its reno-protective potential against HFD and medicines is yet limited.

## Figures and Tables

**Table 1:** Groups’ organization.

Group No.	Individuals/Group (n)	Sex		Euthanized After:	
		Female	Male	2 Months	6 Months
I	16	8	8	8 (4 F + 4 M)	8 (4 F + 4 M)
II	16	8	8	8 (4 F + 4 M)	8 (4 F + 4 M)
III	16	8	8	8 (4 F + 4 M)	8 (4 F + 4 M)
IV	16	8	8	8 (4 F + 4 M)	8 (4 F + 4 M)
V	16	8	8	8 (4 F + 4 M)	8 (4 F + 4 M)
VI	16	8	8	8 (4 F + 4 M)	8 (4 F + 4 M)
VII	16	8	8	8 (4 F + 4 M)	8 (4 F + 4 M)

## Data Availability

All data are contained within the manuscript, but also can be requested from Romeo T. Cristina or Erieg A. Mohamed.

## Supplementary Materials

The following supporting information can be downloaded at: https://www.mdpi.com/article/10.3390/nu16121954/s1, File S1: Assessment of total and individual polyphenols content for Sea buckthorn and organic Grape extract and Oxidative stress markers and antioxidant parameters value.
