# Supplementary material for: The Oxidative Stress Markers’ Protective Influence of Sea Buckthorn and Grape Extracts in Atorvastatin-Treated Hyperlipidemic Rats
Source: Nutrients. 2024 Jun 19;16(12):1954. doi: 10.3390/nu16121954 (PMC11206752; doi:10.3390/nu16121954)
Supplement: Supplementary file 1 [file nutrients-16-01954-s001.zip › nutrients-3038318-supplementary.pdf]

Supplementary file S1

Suporting information 1.

A. Evaluation of the polyphenols content for Sea buckthorn and organic Grape extract

The **total polyphenol content** was determined using the modified Folin-Ciocalteu technique.

Gallic acid (concentration range: 2.5-250 μg/mL) was used to create the calibration curve.

The results were represented in milligrams of **gallic acid equivalent (GAE) / gram** of sample and then converted to milligrams of **GAE/100 g of berry fresh weight**

For identifying and quantifying **individual polyphenols**, LC Shimadzu was used, with SPD-10A UV detectors, and EC 150/2 NUCLEODUR C18 Gravity SB 150 mm × 2.0 mm column, particle size: 5 μm (Macherey-Nagel GmbH & Co. KG, Germany), operating at 20◦C at 0.2 mL/min flow rate.

- Gradient elution of A, aqueous formic acid, and B, acetonitrile and formic acid that was separated the compounds.
- The gradient program was as follows: 5% B (0.01).
- The individual polyphenols were then identified using detectors and EC 150/2 NUCLEODUR C18 Gravity SB 150 mm, 20 min), 5–40% B (20.01–50 min), 40–95% B (50–55 min), and respectively, 95% B (55–60 min).
- The injection volume was set at 20 µL.
- Analysis was carried out at 280 and 320 nm, with the detector set to an acquisition range of 200 to 700 nm.
- Data acquisition, peak integration, and calibrations were performed with LC Solution software from Shimadzu.

For all individual polyphenols employed, calibration curves were prepared in the range of **20–50 µg/mL** (limit of detection 0.4–0.5 µg/mL, limit of quantification 0.6–0.7 µg/mL).

LC Shimadzu showed that, **SBT** contains**:** a **higher concentration** of: rosmarinic acid (43.742 µg/L^{-1}), quercitin (40.534 µg/L^{-1}), resveratrol (28.345 µg/L $^{-1}$), and a **lower concentration** of: kaempferol (6.208 µg/L^{-1}),

Total polyphenols in the Sea buckthorn extract used (µg × mL^{-1})

No.	Compound Name	Retention Time	Area	Height	Concentration
1	Gallic acid	0.000	0	0	0.000
2	Protocatechuic acid	0.000	0	0	0.000
3	Caffeic acid	0.000	0	0	0.000
4	Epicatechin acid	0.000	0	0	0.000
5	*p*-coumaric acid	0.000	0	0	0.000
6	Ferulic acid	0.000	0	0	0.000
7	Rutin	0.000	0	0	0.000
8	Rosmarinic acid	28.851	4,542,389.0	116,917.0	43.742
9	Resveratrol	30.235	4,511,738.0	86,437.0	28.385
10	Quercetin	32.087	2,426,515.0	77,684.0	40.534
11	Kaempferol	33.636	109,323.0	4451	6.208

while the **organic grape extract (Antioxivita)** contains:

higher concentrations of:

- rutin (7.525 μg/L $^{-1}$),
- quercitin (203.798 μg/L $^{-1}$),
- kaempferol (270.556 μg/L $^{-1}$), but

lower levels of:

- rosmarinic acid (26.271 μg/L $^{-1}$) and
- resveratrol (18.615 μg/L $^{-1}$).

Total polyphenols in the organic grape extract used (μg × mL $^{-1}$).

No.	Compound Name	Retention Time	Area	Height	Concentration
1	Gallic acid	0.000	0	0	0.000
2	Protocatechuic acid	0.000	0	0	0.000
3	Caffeic acid	0.000	0	0	0.000
4	Epicatechin acid	0.000	0	0	0.000
5	*p*-coumaric acid	24.945	86,367.0	3501	0.101
6	Ferulic acid	0.000	0	0	0.000
7	Rutin	26.522	997,509.0	24,804.0	7.525
8	Rosmarinic acid	28.466	2,728,108.0	71,428.0	26.271
9	Resveratrol	29.154	2,958,778.0	68,286.0	18.615
10	Quercetin	30.890	12,200,171.0	87,510.0	203.798
11	Kaepferol	34.636	4,764,393.0	36,177.0	270.556

Experimental design & dosage

Group No.	Individuals n = 112	2 Months	6 Months	Dosage (administred by gavage once / day / kg·bw)	
**I**	8F+8M	8	8	Atorvastatin (*Sortis, Pfizer Europe*)	- **20 mg**
**II**	8F+8M	8	8	Atorvastatin + Sea buckthorn:	- **20 mg** + **100 mg**
**III**	8F+8M	8	8	Atorvastatin + Antioxivita:	- **20 mg** + **100 mg**
**IV**	8F+8M	8	8	Antioxivita organic grape extract	- **100 mg**
**V**	8F+8M	8	8	Sea buckthorn extract	- **100 mg**
**VI**	8F+8M	8	8	Control (positive)	- **only** high-fat diet (HFD)
**VII**	8F+8M	8	8	Control (negative)	- **only** rodents normal diet (ND)

Suporting information 2.

B. Spectrophotometric assessment of oxidative stress indicators and antioxidant variables

Malondialdehyde (MDA):

This process depends on the interaction of lipid peroxidation products with thiobarbituric acid (TBA) to release a thiobarbituric acid reactive compound (TBARS). The red compound product was measured at a maximum absorption wavelength of 532 nm using a Tecan Microplate reader (Tecan, Untersberg, Austria GmbH,)

Superoxide dismutase (SOD):

The xanthine and xanthine oxidase reaction systems can produce superoxide anion ($O^{2\cdot}$). The O^2 oxidizes hydroxylamine to form a nitrite that turns purple under the reaction of the developer. SOD can particularly reduce superoxide anion free radicals in the workpiece sample. The absorbance value of the sample tube is lower than that of the control tube due to SOD's inhibitory effect on nitrite formation. The wavelength for detection was 550 nm. For serum samples, 1 SOD activity unit is defined as the quantity of SOD present when the inhibition ratio reaches 50% in 1 mL of solution (U). When the inhibition ratio of 1 mg tissue protein in 1 mL of reaction solution approaches 50%, the concentration of SOD is known as the specific 1 SOD activity unit (U).

Catalase (CAT):

It is an enzyme in organisms that can efficiently and specifically decompose hydrogen peroxide (H_2O_2), in living organisms, CAT is known as a binding enzyme with iron porphyrin as an auxiliary group. Ammonium molybdate can directly prevent or inhibit CAT from decomposing H_2O_2. When residual H_2O_2 interacts with ammonium molybdate, yellowish chemical compounds are generated. The formation of the yellowish chemical compounds at 405 nm can be used to calculate CAT activity. The amount of CAT in 1 mL of serum that decomposes 1 µmol of H_2O_2 per minute at 37 °C is defined as 1 unit. While in tissues, at 37 °C, one unit represents the quantity of CAT in 1 mg of tissue protein that decomposes 1 µmol of hydrogen peroxide per minute

Glutathione peroxidase (GPx):

GPx can stimulate the reaction of hydrogen peroxide following the reduction of glutathione to produce water and oxidized glutathione GSSG. GPx activity can be estimated using the rate of the enzymatic reaction. The consumption of reduced glutathione can be used to estimate glutathione activity.

Because hydrogen peroxide and reduced glutathione can react without GPx catalysis, the quantity of GSH reduction by non-enzymatic reaction should be removed. GSH may combine with dinitrobenzoic acid to form a 5-thio-dinitrobenzoic acid anion, it has a persistent yellow color, and the amount of GPx in 0.1 mL of serum that catalyzes the consumption of 1 µmol/L was measured. GSH is defined as 1 unit after diminishing the effect of a non-enzyme reaction at 37 °C for 5 minutes. In tissue, one unit is defined as the quantity of GPx in 0.1 mg of protein that catalyzes the consumption of one µmol/L GSH. They reduced the effect of the non-enzyme reaction at 37 °C for 5 minutes.

Glutathione reductase converts GSSG to GSH, which can then react with DNTB to yield GSSG and yellow TNB. The total glutathione amount determines the amount of yellow TNB. Total glutathione can be assessed by measuring the optical density value at 412 nm spectrophotometrically. The concentration of GSSG was determined by first removing GSH from the specimen that used a suitable reagent; the best detection wavelength at 25 °C was 412 nm. At 412 nm, total glutathione was measured spectrophotometrically. Calculating GSSG from total glutathione yielded a reduced glutathione concentration. The serum results are presented as µmol/L, while the tissue sample results are expressed as µmol/g.

Total Antioxidant Capacity (TAC):

A system's total antioxidant components and enzymes can eliminate all forms of harmful substances, as well as control oxidative stress induced by ROS. The total amount indicates the system's TAC. Many antioxidants in the organism can convert Fe^{+3} to Fe^{+2} which leads to forming stable compounds with phenanthroline compounds The absorbance at 520 nm can be used to compute the TAC. The optical density value of the reaction system increased by 0.01 by 1 mL of sample/minute at 37 °C, referring to a unit of TAC.

Suporting information 3.

C. Oxidative stress markers and antioxidant parameters value.

Table S1. The activity of **serum oxidative stress markers** and antioxidant indicators in the experimental groups during experimental period

Group	MDA nmol×mL^{-1}	T-SOD U×mL^{-1}	CAT U×mL^{-1}	GPx µmol×L^{-1}
**After: Two months**				
I	22.266 ±2.787	270.543 ±5.745	13.468 ±4.179	4450.349 ±72.204
II	15.393 ±1.444	258.188 ±3.424	11.633 ±3.040	4116.281 ±288.967
III	17.332 ±1.873	266.653 ±6.033	16.335 ±2.747	4621.399 ±76.432
IV	23.602 ±2.044	262.74 ±8.607	14.244 ±5.319	4623.072 ±118.017
V	16.8 ±3.273	283.472 ±6.341	10.641 ±0.849	4028.686 ±30.884
VI	25.837 ±2.270	271.916 ±5.472	10.005 ±2.598	4054.79 ±129.634
VII	11.771 ±1.253	267.001 ±7.022	12.201 ±2.108	3973.647 ±53.649
**After: Six months**				
I	25.498 ±1.584	257.365 ±17.026	21.714 ±2.653	4222.421 ±207.109
II	12.371 ±1.530	270.711 ±12.913	13.983 ±1.453	4093.727 ±109.172
III	13.452 ±1.171	270.417 ±10.407	22.676 ±2.240	4575.593 ±74.793
IV	17.978 ±1.481	269.061 ±8.851	15.264 ±2.769	4329.351 ±79.528
V	11.933 ±1.333	268.328 ±3.209	16.201 ±1.114	4047.31 ±74.555
VI	30.853 ±2.657	257.727 ±7.658	26.051 ±3.025	5319.703 ±87.212
VII	16.786 ±1.228	259.422 ±6.925	16.520 ±1.516	4022.37 ±165.621

Table S2. The activity of the **serum T-GSH/GSSG** and **TAC values** in experimental groups during experimental period

Group	T-GSH µmol×L^{-1}	GSSG µmol×L^{-1}	GSH µmol×L^{-1}	TAC U×mL^{-1}
**After: Two months**				
I	0.830 ±0.027	0.276 ±0.023	0.277 ±0.052	4.764 ±0.132
II	1.180 ±0.108	0.199 ±0.036	0.780 ±0.058	6.329 ±0.304
III	1.104 ±0.140	0.124 ±0.017	0.855 ±0.124	4.607 ±0.260
IV	1.277 ±0.095	0.187 ±0.040	0.901 ±0.083	5.087 ±0.179
V	2.399 ±0.099	0.068 ±0.006	2.262 ±0.096	5.214 ±0.250
VI	1.586 ±0.102	0.131 ±0.016	1.323 ±0.107	5.612 ±0.893
VII	0.986 ±0.053	0.255 ±0.022	0.476 ±0.071	4.875 ±0.305
**After: Six months**				
I	0.751 ±0.071	0.223 ±0.017	0.305 ±0.060	5.605 ±0.482
II	0.793 ±0.061	0.065 ±0.018	0.662 ±0.039	6.174 ±0.457
III	0.954 ±0.056	0.130 ±0.023	0.694 ±0.025	5.396 ±0.469
IV	2.141 ±0.110	0.159 ±0.054	1.822 ±0.168	4.751 ±0.499
V	2.305 ±0.108	0.169 ±0.008	1.965 ±0.112	6.032 ±0.395
VI	0.703 ±0.029	0.199 ±0.014	0.304 ±0.031	2.926 ±0.224
VII	1.014 ±0.050	0.281 ±0.032	0.452 ±0.045	5.786 ±0.259

Table S3. The values of the **liver** lipid peroxidation and antioxidant indicators in the experimental groups following six month treatment.

Group	MDA µmol×g.prot^{-1}	T-SOD U×mg.prot^{-1}	CAT U×mg.prot^{-1}	GPx U×mg.prot^{-1}
I	2.602 ±0.332	743.9 ±76.94	383.7 ±27.68	20330 ±1964
II	1.547 ±0.095	668 ±21.17	486.1 ±42.27	22087 ±1414
III	1.354 ±0.114	622.9 ±68.08	463 ±38.29	18206 ±1526
IV	1.741 ±0.088	758 ±43.52	571.2 ±36.6	21434 ±1541
V	1.39 ±0.246	745.1 ±63.27	564.1 ±116.3	20216 ±976.9
VI	5.874 ±1.806	571.7 ±60.61	581.6 ±65.99	20864 ±1306
VII	1.256 ±0.170	659.2 ±23.46	390.4 ±59.04	19632 ± 994.7

Table S4. The values of the **liver** T-GSH/GSSG in the experimental groups following six-month treatment.

Group	T-GSH µmol×g.prot^{-1}	GSSG µmol×g.prot^{-1}	GSH µmol×g.prot^{-1}
I	4.746 ±0.126	0.432 ±0.038	3.88 ±0.178
II	4.756 ±0.065	0.500 ±0.056	3.755 ±0.060
III	4.697 ±0.080	0.436 ±0.057	3.824 ±0.170
IV	4.74 ±0.029	0.464 ±0.025	3.811 ±0.034
V	4.754 ±0.035	0.554 ±0.068	3.639 ±0.107
VI	4.807 ±0.060	0.367 ±0.104	4.072 ±0.187

**VII**	4.689 ±0.021	0.616 ±0.038	3.45 ±0.091

Table S5. The values of the **kidney** lipid peroxidation and antioxidant indicators in the experimental groups following six-month treatment.

Group	MDA μmol×g.prot^{-1}	T-SOD U×mg.prot^{-1}	CAT U×mg.prot^{-1}	GPx U×mg.prot^{-1}
**I**	3.25 ±0.200	270.1 ±14.72	111.2 ±16.62	4600 ±454
**II**	3.462 ±0.295	284.9 ±9.988	122.9 ±13.2	5807 ±1172
**III**	2.464 ±0.137	294.4 ±18.6	74.19 ±13.47	4092 ±712.9
**IV**	2.791 ±0.196	267.8 ±21.87	64.2 ±6.763	6242 ±1140
**V**	2.475 ±0.113	285.5 ±15.48	79.57 ±16.51	4759 ±1390
**VI**	2.752 ±0.314	354 ±43.76	71.34 ±4.987	4234 ±1092
**VII**	2.925 ±0.226	351.2 ±13.9	61.86 ±8.597	9592 ±527.3

Table S6. The values of the **kidney** T-GSH/GSSG in the experimental groups following six-month treatment.

Group	T-GSH μmol×g.prot^{-1}	GSSG μmol×g.prot^{-1}	GSH μmol×g.prot^{-1}
**I**	0.750 ±0.027	0.091 ±0.003	0.566 ±0.034
**II**	0.874 ±0.044	0.106 ±0.002	0.662 ±0.040
**III**	0.875 ±0.041	0.102 ±0.012	0.670 ±0.030
**IV**	0.889 ±0.027	0.100 ±0.009	0.702 ±0.017
**V**	0.585 ±0.014	0.069 ±0.001	0.446 ±0.018
**VI**	0.875 ±0.021	0.118 ±0.008	0.639 ±0.013
**VII**	0.605 ±0.033	0.087 ±0.002	0.43 ±0.030
